# The Use of the Phrenic Nerve Communicating Branch to the Fifth Cervical Root for Nerve Transfer to the Suprascapular Nerve in Infants with Obstetric Brachial Plexus Palsy

**DOI:** 10.1155/2014/153182

**Published:** 2014-03-31

**Authors:** M. M. Al-Qattan, A. A. F. El-Sayed

**Affiliations:** The Division of Plastic Surgery and Department of Obstetrics & Gynecology, King Saud University, P.O. Box 18097, Riyadh 11415, Saudi Arabia

## Abstract

Traditionally, suprascapular nerve reconstruction in obstetric brachial plexus palsy is done using either the proximal C5 root stump or the spinal accessory nerve. This paper introduces another potential donor nerve for neurotizing the suprascapular nerve: the phrenic nerve communicating branch to the C5 root. The prevalence of this communicating branch ranges from 23% to 62% in various anatomical dissections. Over the last two decades, the phrenic communicating branch was used to reconstruct the suprascapular nerve in 15 infants. Another 15 infants in whom the accessory nerve was used to reconstruct the suprascapular nerve were selected to match the former 15 cases with regard to age at the time of surgery, type of palsy, and number of avulsed roots. The results showed that there is no significant difference between the two groups with regard to recovery of external rotation of the shoulder. It was concluded that the phrenic nerve communicating branch may be considered as another option to neurotize the suprascapular nerve.

## 1. Introduction

Sacrifice of the phrenic nerve in healthy adults does not result in any clinically significant problems. Hence, the entire phrenic nerve is commonly sacrificed and used for nerve transfer in traumatic brachial plexus injuries in adults [1–4]. In contrast, injury to the phrenic nerve during brachial plexus surgery in infants is expected to the result in postoperative clinically significant problems in about 30% of cases [5]. These problems include respiratory distress (which may require intubation), recurrent lower respiratory tract infections, lung collapse, and pneumonia [5]. Hence, the use of phrenic nerve transfer in infants is generally not recommended.

**Figure 1:**
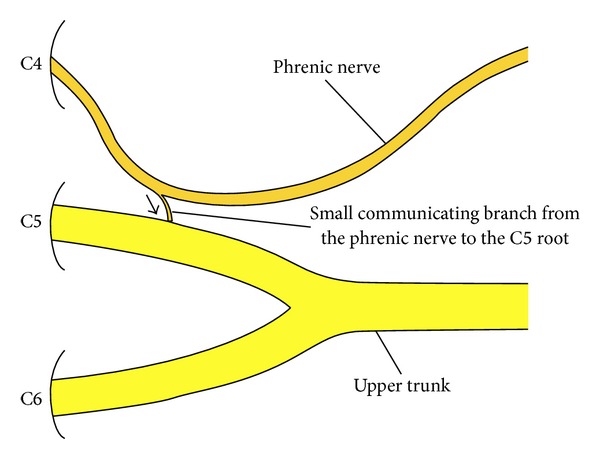
A diagram of the communicating branch from the phrenic nerve to the C5 root.

The anatomy of the phrenic nerve is well described. The phrenic nerve takes origin mainly from the fourth cervical (C4) root. In its early course close to its origin, the nerve may give a communicating branch to the C5 root (Figure 1) [6]. This communicating branch represents the contribution of the C4 root to the brachial plexus. Several anatomists studied this phrenic nerve communicating branch in cadaveric dissections [7, 8]. The prevalence of this communicating branch ranges from 23% to 62% in various dissections. The fibers of this communicating branch eventually give contributions to different nerves of the brachial plexus including the suprascapular, lateral pectoral, musculocutaneous, subscapular, axillary, and radial nerves. Finally, the size of the phrenic nerve communicating branch to the C5 root is variable. To the best of the author's knowledge, the use of this communicating branch for nerve transfer has not been previously reported. The current paper reports on the results of phrenic nerve communicating branch transfer for suprascapular nerve reconstruction in 15 infants with obstetric brachial plexus palsy and compares these results to the results of the spinal accessory to suprascapular nerve transfer in another 15 infants with obstetric palsy.

## 2. Patients and Methods

This retrospective study was approved by the Research Committee of the Department of Surgery at our hospital. The data of our multidisciplinary obstetric brachial plexus clinic was reviewed over the last two decades (1994–2013 inclusive). Only patients who underwent primary brachial plexus exploration and who have adequate data and minimum follow-up of 2.5 years were included. There were a total of 15 cases in whom the phrenic communicating branch was used to neurotize the suprascapular nerve (Group I). Another 15 cases in which the accessory nerve was used to neurotize the suprascapular nerve were selected to match the former 15 cases with regard to age at the time of surgery, type of palsy, and number of avulsed roots (Group II). The demographic data of the cases in both groups are shown in Table 1.

### 2.1. Surgical Technique

Identification of the phrenic nerve is done as per the technique of Al-Qattan [9]. The supraclavicular nerves are identified at the clavicular incision and followed to their origin from the fourth cervical (C4) root. This will identify the phrenic nerve which also arises from the C4 root. We normally do not divide the communicating branch from the phrenic nerve to the C5 root. However, the communicating branch is identified and used for transfer in cases of C5 root avulsion or proximal C5 root rupture near the foramen. One sural nerve cable graft is used to connect the phrenic communicating branch to the prepared suprascapular nerve stump. Coaptation is done using fibrin glue.

Identification of the spinal accessory nerve is done as per the technique of Al-Qattan and El-Shayeb [10]. The nerve is transected just distal to the branch to the upper part of the trapezius muscle. Neurotization of the suprascapular nerve is done either directly or via a sural nerve graft. Coaptation is done using fibrin glue.

### 2.2. Assessment of External Rotation of the Shoulder

Assessment of external rotation of the shoulder was done in all patients prior to surgery and at final follow-up (prior to performing any secondary procedures to the shoulder). True glenohumeral external rotation (with the elbow flexed and the arm adducted) was measured in degrees as described by Pondaag et al. [11]. Functional external rotation of the shoulder was graded as per Al-Qattan's modification of the Mallet Score for external rotation [12] as shown in Table 2.

### 2.3. Statistical Analysis

Comparison between the two groups with regard to the glenohumeral external rotation measurements was done using the Mann-Whitney test. Functional external rotation results of the two groups were compared using either the Fisher exact or the chi-square tests. *P* values less than 0.05 were considered significant.

## 3. Results

Prior to the neurotization procedure, none of the infants had active external rotation of the shoulder. At final follow-up (range 2.5–4.5 years with a mean of 3 years and 2 months) after surgery, the outcomes of external rotation of the shoulder with regard to measurements of true glenohumeral external rotation and functional external rotation are shown in Tables 3 and 4, respectively.

Data in Table 3 are arranged in a decreasing order of measurements for both groups. Only 5 patients in each group had an external rotation over 20°. The means (SD) were 27.0° (28.5°) and 24.7° (28.0°) for Groups I and II, respectively. There were 4 complete failures in the phrenic nerve group (Group I) and 5 complete failures in the accessory nerve group (Group III). The Mann-Whitney test showed no significant difference (*P* = 0.7) between the measurements of the two groups.

Table 4 shows the functional results. None of the patients had a Grade 5 result. The percentages of children in the remaining grades (Grades 1–4) were similar in both groups with no significant differences (*P* values more than 0.5; see Table 4) between the two groups, using Fisher/chi-square tests.

Functionally, many children were able to compensate (using other movements) for the deficiency in glenohumeral external rotation to reach the mouth, ear, and occiput. Compensatory mechanisms included shoulder abduction (also known as the trumpet posture), thoracoscapular movements, curative of the spine, and bending of the head. Hence, 80% of children in Group I and 73% of children in Group II were able to reach the mouth.

## 4. Discussion

The suprascapular nerve supplies both the supraspinatus (which contributes to shoulder abduction) and infraspinatus (the primary shoulder external rotator) muscles. The main aim of reconstruction of the suprascapular nerve is to improve active external rotation of the shoulder. Traditionally, neurotization of the suprascapular nerve in obstetric palsy is done using either the proximal C5 root stump or the accessory nerve [13–18]. Several authors found no significant differences in external rotation of the shoulder after suprascapular nerve reconstruction using either of these two traditional techniques [11, 19]. Our study introduces the phrenic nerve communicating branch as another potential donor for neurotization of the suprascapular nerve and demonstrates that the results are similar to neurotization using the accessory nerve. It is important to note that the phrenic nerve communicating branch is not always available for neurotization because it may be absent or too small. Furthermore, if the neuroma of the C5 root is distal to the communicating branch, we do not normally divide the communicating branch. Another disadvantage of using the communicating branch is the need for a nerve graft in all cases. In contrast, direct neurotization may be possible when using the accessory nerve.

The overall results of suprascapular nerve reconstruction varied in different series of obstetric palsy. One reason for this variation is the way the results are presented. As we demonstrated in our series as well as in the series of Pondaag et al. [11], the results of functional external rotation of the shoulder are generally better than measurements of active external rotation in degrees because of compensatory mechanisms. Another reason is the technique of measurement of active external rotation. “True” glenohumeral external rotation should be measured with the arm adducted. Schaakxs et al. [18] demonstrated that the results will be better when active external rotation of the shoulder is measured with the arm in abduction compared to adduction, and the authors attributed this to the presence of cocontractions.

Almost one-third of our patients did not recover any active external rotation of the shoulder following suprascapular nerve reconstruction (see Table 3). Similar results were reported by Pondaag et al. [11]. One reason for complete failures may be the double level injuries (also known as the double crush) to suprascapular nerve at the level of the neuroma as well as the level of the suprascapular notch. Other factors that could affect the overall results include the age at the time of surgery, the type of palsy, the number of avulsed roots, the quality of proximal stump of the donor nerve, and the use of intervening nerve graft. In our series, we tried to minimize these factors by selecting Group II patients to match Group I with regard to age, type of palsy, and the number of avulsed roots. Surgeons tend to dissect the spinal accessory nerve too distally to be able to do direct neurotization of the suprascapular nerve, and the more distal the dissection, the less the number of available axons in the proximal stump. The phrenic nerve communicating branch should only be used when it is of good size (to match the size of the suprascapular nerve) and the results may be downgraded by the use of nerve grafts. However, it should be considered as one of the options of neurotization especially in cases with multiple root avulsions.

## Figures and Tables

**Table 1:** Demographic data of 30 infants who underwent neurotization of the suprascapular nerve using either the phrenic nerve communicating branch (*n* = 15) or the spinal accessory nerve (*n* = 15).

	Phrenic nerve communicating branch (*n* = 15)	Spinal accessory nerve (*n* = 15)
Male/female	7/8	8/7
Right/left sided palsy	10/5	9/6
Mean age at the time of neurotization	4.1 months	4.3 months
Erb's/total palsy	9/6	9/6
Average number of avulsed roots	1.5	1.5

**Table 2:** Assessment of functional external rotation of the shoulder.

Grade	Description
1	The hand reaches the abdomen/thorax
2	The hand reaches the mouth
3	The hand reaches the ear
4	The hand reaches the occiput
5	Normal power and range of motion

**Table 3:** True glenohumeral external rotation following neurotization of the suprascapular nerve.

Case number	True glenohumeral external rotation following neurotization of the suprascapular nerve using either	
	Phrenic communicating branch (*n* = 15)	Accessory nerve (*n* = 15)
1	90°	85°
2	70°	75°
3	60°	50°
4	40°	40°
5	40°	35°
6	40°	35°
7	20°	20°
8	15°	10°
9	10°	10°
10	10°	10°
11	10°	0
12	0	0
13	0	0
14	0	0
15	0	0

“0” means that there is no active external rotation.

**Table 4:** Functional external rotation of the shoulder following neurotization of the suprascapular nerve.

Functional external rotation of the shoulder	Group I: phrenic nerve group (*n* = 15)	Group II: accessory nerve group (*n* = 15)
	Number of children (%) in each grade	Number of children (%) in each grade
Grade 1: the hand reaches the abdomen/thorax	3 (20.0%)*	4 (26.7%)*
Grade 2: the hand reaches the mouth	2 (13.3%)**	2 (13.3%)**
Grade 3: the hand reaches the ear	4 (26.7%)***	3 (20.0%)***
Grade 4: the hand reaches the occiput	6 (40.0%)****	6 (40.0%)****
Grade 5: normal power and range of motion	0	0

By Fisher Exact test: *P = 0.5, **P = 0.7, ***P = 0.5.

By chi-square test: ***P = 0.99.
